# Beyond the Bench: An Integrative Curriculum: Science by Design

**DOI:** 10.1289/ehp.114-a525

**Published:** 2006-09

**Authors:** Tanya Tillett

With our increased awareness of the need to understand human–environment interactions, it is more critical than ever to spark and nourish children’s interest in science. Now an NIEHS-sponsored program at the Baylor College of Medicine Center for Educational Outreach is responding to this educational challenge by offering the ECOS (Environment as a Context for Opportunities in Schools) project, a teacher professional development and curriculum implementation project with an integrative approach that focuses on strengthening science teaching and learning at the elementary school level.

Established in 2000, the ECOS project was created by a team of educators, scientists, and health specialists to connect environmental health science with health, reading/language arts, mathematics, and social studies for Houston-area elementary schools. Two distinct educational series for grades K–2 and 3–5, developed previously with funding from the NIEHS and the National Center for Research Resources, were first implemented in six schools. Later, a charter school and four more elementary schools were added to the project. Both series of curriculum materials include interactive class lessons and engaging adventure storybooks illustrated by T. Lewis, co-creator of the syndicated comic strip and animated movie *Over the Hedge*.

“My World and Me,” the module developed for students in grades K–2, contains two units, “Living Things and Their Needs” and “Resources and the Environment,” each with 10 sequential series of lessons, an illustrated storybook for each student that teaches science and health concepts while relating the adventures of Tillena Lou Turtle, and a “read aloud” big book for classroom use. An accompanying teacher’s guide to hands-on activities stresses inquiry-based lessons such as observing an earthworm, identifying its needs, and building an appropriate habitat in a terrarium made of a plastic soda bottle.

The “My World” (formerly “My Health, My World”) series has four units on integrative topics for students in grades 3–5: “Air,” “Water,” “Global Resources,” and “Food.” Each unit includes a story-book featuring the adventures of squirrels Riff and Rosie, an *Explorations* mini-magazine for each student to share with family members, an activity guide for teachers, and supplements (for reading/language arts and mathematics) related to the storybook. Typical language arts activities include “finding the main idea” and writing about cause-and-effect relationships. Math activities focus on developing basic skills using science information related to a specific unit—for example, estimating metric measures, solving number puzzles, and creating and using graphs.

The program also provides support and training for teachers to help maximize the effectiveness of the curriculum. New teacher enrollees receive two days of professional development including an overview of the curriculum content, a complete package of instructions, classroom activity kits, and training in conducting the different activities in the classroom units. Those already participating in the program receive additional training in enrichment activities scheduled several times throughout the academic year.

Both series are designed to be flexible and accommodate a variety of teaching methods and styles; the program designers note that schools have begun customizing unit activities to fit their own educational needs and priorities. Nancy Moreno, associate director of the Center for Educational Outreach and the ECOS project principal investigator, says customization shows that teachers are actively involved in the planning process for the project’s implementation in their schools, and are intellectually invested in it. For example, she describes how the “Water” unit, initially designed for use in 2nd grade to meet science standards for that grade level, is now being used in 4th-grade classes in some schools because it better fits requirements for that grade level. “Since the materials are not grade level–specific, teachers can adjust them up or down by using suggestions and extensions that are provided in the teacher’s guide,” she explains.

ECOS project participation is also having a positive impact on overall science learning for both students and teachers. The program developers have measured student knowledge using tools such as pre- and post-tests and essay assignments, and have observed a noticeable increase in student performance, especially among Spanish-speaking students, at all grade levels. Participating teachers have also shown gains in content knowledge when taking similar types of assessments.

“The ECOS project is an example of how scientific research institutions can collaborate effectively with local schools to improve science teaching and learning,” says Moreno. “Shortcuts do not work. However, when scientists, science educators, school administrators, and classroom teachers work together, the nature of science instruction received by students changes profoundly. The payoffs from this approach are beginning to be visible in the ECOS project.”

More information on the project, including ordering information, is available at http://www.ccit.bcm.tmc.edu/ceo/. The project, which has received such honors as finalist status for the State of Texas Environmental Excellence Award, is developing new units to integrate science inquiry into schoolwork. Coming up in the next school year is a unit for 5th graders that focuses on alcohol as a chemical that can interact with the human body.

## Figures and Tables

**Figure:**
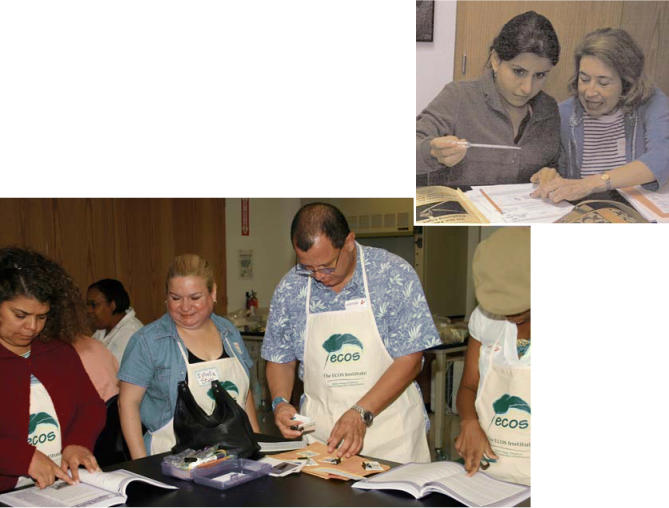
Teaching the teachers ECOS participants learn to implement curricula that examine topics such as the processes involved in creating everyday objects from natural resources (large photo) and the physical properties of water (inset).

